# Efficiency of botulinum toxin injection into the arm on postural balance and gait after stroke

**DOI:** 10.1038/s41598-023-35562-1

**Published:** 2023-05-24

**Authors:** Junhee Lee, Ji Eun Park, Byung Heon Kang, Seung Nam Yang

**Affiliations:** 1 Department of Physical Medicine and Rehabilitation, Korea University Guro Hospital, 148 Gurodong-Ro, Guro-Gu, Seoul, 08308 Republic of Korea; 2 Department of Physical Medicine and Rehabilitation, Korea University College of Medicine, 73, Goryeodae-Ro, Seongbuk-Gu, Seoul, 02841 Republic of Korea

**Keywords:** Diseases, Medical research, Neurology

## Abstract

The purpose of this study was to clarify the association between improvement of spasticity in hemiplegic patient’s upper extremity with Botulinum toxin injection and improvement in postural balance and gait function. For this prospective cohort study, sixteen hemiplegic stroke patients with upper extremity spasticity were recruited. The plantar pressure with gait parameters, postural balance parameters, Modified Ashworth Scale, and Modified Tardieu Scale were evaluated before, 3 weeks and 3 months after Botulinum toxin A (BTxA) injection. Spasticity of hemiplegic upper extremity before, and after BTxA injection were significantly changed. Plantar pressure overload in affected side was reduced after BTxA injection. The mean X-speed and the horizontal distance decreased in postural balance analysis with eyes-opened test. Improvement in hemiplegic upper extremity spasticity showed positive correlation with gait parameters. In addition, improvement in hemiplegic upper extremity spasticity was positively correlated with change in balance parameters in postural balance analysis with eyes-closed and dynamic tests. This study focused on the effect of stroke patient’s hemiplegic upper extremity spasticity on their gait and balance parameters and identified that the BTxA injection on hemiplegic patient’s spastic upper extremity improve postural balance and gait function.

## Introduction

Spasticity is a common complication after a stroke. Approximately, 19–43% of stroke patients experience spasticity^1–4^ as an aspect of upper motor neuron disease characterized by increased muscle tone, tendon reflexes, and spasms^5^. In particular, spasticity of the upper extremities worsens the impairment of functional ability over a long period of time. The prevalence of upper-extremity spasticity at 12 months post-stroke varies from 17 to 38%^6–8^. Among patients with upper-extremity spasticity, 46% experience deterioration of arm function^9^.

Botulinum toxin (BTx) is a popular treatment for spasticity. The limited permeability of BTx on blood–brain barrier and the mechanism of therapeutic effect on central nervous system (CNS) has been issued^10,11^. Therefore, peripheral BTx injection has been focused on its effect on CNS in both direct and indirect way^12,13^. Indirect central effect was explained that BTx may influence the central sensorimotor integration with altered peripheral mechanism. Direct central effect was suggested from retrograde axonal transport of BTx from the injection site to CNS^14,15^.

Previous studies have suggested that BTx injection into the spastic lower extremities may lead to improvement in balance and gait function^16–22^. They showed significant improvements in various spatio-temporal parameters, kinematic and kinetic measurements during gait after BTx injection. Also, upper extremity BTx injection have add-on effect on patients’ truncal balance control as well as improvement of upper extremity spasticity^23,24^. Improvement of truncal balance can have positive effect on the improvement of gait function. However, there are insufficient studies to clarify this phenomenon. Some trials have determined the effect of improvement in upper-extremity spasticity on changes in balance and gait control. Hirsch et al. suggested an association between upper-extremity BTx injection and stride time and ankle and knee range of motion (ROM) of the paretic leg in stroke^25^. The authors reported that injection of BTx reduced stride time in all stroke patients. In addition, when participants were stratified according to fast or slow stride time, a pronounced effect on ankle and knee ROM in slow-striding participants was observed. Esquenazi et al. showed that treatment of elbow flexor spasticity with BTxA injection can improve gait disturbance with increasing walking velocity in patients with upper-motor neuron syndrome^26^. Previous researches tried to find out the relation between upper-extremity spasticity and functional capacity with spatio-temporal parameters and conventional ROM measurements. And there was no previous analysis to prove the correlation of these two components.

We hypothesized that improvement in spasticity in the upper extremities of hemiplegic patients by BTx injection would improve postural balance and gait function. In order to establish a clear relationship, we investigate the correlation between changes in quantitative evaluation of postural balance and gait function and changes in upper limb spasticity before and after botulinum toxin injection.

## Methods

### Participants

This study was prospective cohort study. A total of 16 post-stroke patients with hemiplegia were recruited from the Department of Physical Medicine and Rehabilitation, Korea University Guro Hospital, between March 1, 2020, and May 31, 2021, for this study. Eleven patients were male and 5 were female. The inclusion criteria were (1) age > 18 years, (2) at least 6 weeks after stroke diagnosis, (3) upper-extremity (elbow, wrist and finger) spasticity Modified Ashworth Scale (MAS) score > 2, and (4) ability to stand and walk safely without help or assistance. The exclusion criteria were as follows: (1) improper indication for botulinum toxin A (BTxA) injection, for example, myasthenia gravis, Eaton-Lambert syndrome, amyotrophic lateral sclerosis, and motor neuropathy; (2) previous contracture and/or deformity of the upper extremities; (3) concurrent peripheral neuropathy and/or myopathy; and (4) difficulty in participating in the study due to cognitive impairment.

There were 12 patients who had never received a BTx injection before this study and 4 patients who had received BTx before this study. Those 4 patients were recruited at least 6 months from BTx injection. All participants continued their previously scheduled oral medication and rehabilitation.

### Botulinum toxin intervention

Appropriate arm muscles for BTxA injection were clinically selected by physical examination. In our study, we used Nabota^®^ (Daewoong Pharmaceutical Co. Ltd., Seoul, Korea) as a BTx agent. It is a protein of high purity and quality obtained from the natural strain *Clostridium botulinum* (type A)^27^. BTxA was diluted with 0.9% sodium chloride solution and injected into the bellies of the upper-extremity muscles. Muscles to be injected were determined individually, according to patient’s condition. The toxin dose was established for each patient: it ranged between 80 and 300 unit. From 1 up to 3 local points of injections considering muscle size have been selected. Intramuscular injections with electrostimulation guidance was performed once by a physiatrist with 20 years of clinical experience in stroke-related spasticity. Participants continued their routine schedule of medications and rehabilitation programs and were required to observe any considerable changes during the study.

### Spasticity evaluation and functional measurement

All participants were assessed at baseline (pre-intervention), three weeks after intervention (post-intervention), and three months after intervention (follow-up) with BTxA. The MAS and Modified Tardieu Scale (MTS) were used to assess the degree of spasticity. Also, the hemiplegic upper extremities were evaluated using the Fugl-Meyer Assessment (FMA) and Action Research Arm Test (ARAT). In addition, the hemiplegic lower extremities were evaluated using the FMA. Furthermore, functional ambulatory category (FAC) was estimated.

### Plantar foot pressure analysis

In every session, plantar pressure with center of pressure (CoP) excursion was analyzed using an insole pressure measurement system (Medilogic^®^, T&T medilogic Medizintechnik GmbH, Unterschleissheim, Germany)^28,29^. In our laboratory, different-sized insoles were used, consisting of resistive sensors based on size. The sensor density was 0.79/cm^2^, and the sensor could withstand a maximum pressure of 64 N/cm^2^. The plantar pressure data were collected at a sampling frequency of 60 Hz and transmitted by cables from the insoles to the analog–digital box worn at the participant’s waist. The data were transmitted from the analog–digital box to a computer through a wireless connection^30^.

A static standing trial was conducted to confirm appropriate positioning of the insole, followed by a dynamic walking trial. All patients walked at least 5 m with normal gait and more than 12 steps without any personal assistance or assistive devices. Gait speed was determined for each participant in a comfortable and tolerable range. Dynamic measurements were conducted for at least six trials, and the most stable and best-performing trial was selected. Pressure load (%), and spatiotemporal parameters (gait speed (m/s), stride length (m), stance and swing phase duration (s)) were measured.

### Postural balance measurement

A computer-based force platform test (Good Balance, Metitur Ltd,. Finland, www.metitur.com) was used to evaluate postural balance^31,32^. This system consisted of an equilateral triangular force platform (width 800 mm, height 70 mm, with strain gauge transducers at each corner of the platform), a three-channel direct current amplifier, an eight-channel 12-byte analog-to-digital converter (sampling frequency 50 Hz), and a program installed on a laptop computer.

Based on the vertical force signals from each corner of the platform, the system calculated the X (mediolateral, ML) and Y (anteroposterior, AP) coordinates of the CoP affecting the platform while the patients were standing on it. Based on the coordinate values for X and Y, various balance parameters such as mean speed of the movement of the CoP in the ML direction (mean X-speed (mm/s)), mean speed of the movement of the CoP in the AP direction (mean Y-speed (mm/s)), performing time (s), full distance of CoP movement (total distance (mm)), distance of the ML direction made by the CoP (horizontal distance (mm)) and distance of the AP direction made by the CoP (vertical distance (mm)) were calculated^33^.In every test, the participants stood on the point with the hindfoot of both feet located at center ‘O’ mark of the triangular force platform and both feet 3 cm apart. Additionally, both ankles were externally rotated by 15°. Balance was tested in three different conditions: (1) In the eyes-opened test, the participant was in the normal standing position, feet slightly apart, arms in a relaxed position, and gaze fixed on a computer monitor (test duration was 30 s). (2) In the eyes-closed test, all conditions were the same as those in the eyes-opened test. (3) In the dynamic test, the participants were instructed to perform a specific movement task presented on the monitor. Evaluations for each of the three conditions were conducted for at least two trials, and the trial with the best performance was selected.

### Statistical analysis

Descriptive statistics were used to assess the general characteristics and demographic factors of the patients. Friedman’s test was employed to detect differences in upper-extremity spastic measurements, spatiotemporal gait parameters, and calculated coordinates of the CoP at pre-intervention (baseline), post-intervention, and follow-up evaluation. The Wilcoxon signed-rank test was computed to determine the difference between pre- (baseline) and post-injection. Spearman’s rho was used to analyze the correlation between improvement in spastic measurements, foot pressure, and postural balance parameters. SPSS version 26.0 software (SPSS Inc., Chicago, IL, USA) was used for all analyses, with the statistical significance level set at *p* < 0.05.

### Ethics

All patients recruited for this study provided written consent. The participants were informed prior to evaluation that the collected data could only be used for study purposes, and they had the right to refuse this use at any time. The study was approved by the institutional review board of the Korea University Guro Hospital (2019GR0159), and was conducted in accordance with the Declaration of Helsinki. To increase the quality of reporting of this observational study, STROBE guidelines were followed^34^. All participants signed an informed consent form before participating in the study.

## Results

Sixteen patients were enrolled in this study. Patient characteristics, demographic factors, target muscle with exact BTxA dosage, and baseline upper extremities evaluations are described in Table 1. The average age of the participants was 50.8 ± 12.3 years. Eight patients had hemorrhagic stroke and 8 had ischemic stroke, while 6 had right hemiplegia and 10 had left hemiplegia. The average upper extremity FMA score was 18.6 ± 15.8, and the average ARAT score was 15.6 ± 15.3. The average lower extremity FMA score was 18.6 ± 8.0, and the average FAC was 3.9 ± 0.6.

**Table 1:** Subject characteristics.

Subject no.	Age (years)	Sex	BMI	Time since stroke (months)	Stroke type	Affected side (L/R)	Total unit injected	Muscles injected^1^
1	30	M	30.2	159	H	L	270	1–6
2	59	M	22.5	4	I	R	170	1–3
3	40	M	23.8	23	H	L	300	1–6
4	62	M	22.0	39	I	L	300	1–6
5	62	M	28.7	39	H	L	300	1–7
6	65	F	23.9	54	I	R	250	1–6
7	63	M	24.9	33	H	R	230	2–4, 6, 7
8	34	M	28.3	46	I	L	300	1–4, 6, 7
9	42	F	21.7	318	H	L	240	1–4, 6, 7
10	65	F	23.9	3	I	L	110	1, 2, 5
11	53	M	24.5	33	I	L	80	2, 3
12	62	F	24.8	18	H	L	160	1–3, 6
13	35	M	29.1	21	H	L	170	1–3, 6
14	47	M	22.6	10	I	R	220	1–3, 5
15	52	M	25.2	5	I	R	300	2–7
16	42	F	33.6	25	H	R	250	2–4, 6, 7

^1^Muscle injected: 1, biceps brachii; 2, brachioradialis; 3, pronator teres; 4, flexor carpi radialis; 5, flexor carpi ulnaris; 6, flexor digitorum profundus; 7, flexor digitorum superficialis.

Abbreviations: *BMI* body mass index, *I* ischemic, *H* hemorrhagic, *L* left, *R* right.

### Effect of BTxA injection on spasticity in affected upper extremities

Compared to the pre-injection evaluation, the MAS grades of the elbow extensor and flexor, wrist flexor, finger extensor, and flexor were significantly decreased at post-injection and follow-up. The MTS (R2-R1) of the elbow flexor and wrist extensor was significantly changed, as shown in Table 2.

**Table 2:** Changes in MAS and tardieu scale.

Spasticity parameters			Pre-injection	Post-injection (3 weeks later)	Follow up (3 months later)	*P* value	
						*P*^1^ (pre- post)	*P*^2^
MAS	Elbow	Extensor	2.0 (1.0–2.75)	1.0 (0–1.75)	1.0 (0–2.25)	0.016*	0.016*
		Flexor	2.0 (1.25–3.0)	1.0 (0.25–1.0)	1.0 (1.0–2.0)	0.001*	< 0.001*
	Wrist	Extensor	1.0 (0–2.75)	0 (0–1.0)	0 (0–1.0)	0.041*	0.076
		Flexor	3.0 (3.0–3.0)	1.0 (1.0–1.0)	1.0 (0–1.25)	0.001*	< 0.001*
	Finger	Extensor	1.0 (0–3.0)	0 (0–1.0)	0 (0–0)	0.026*	0.003*
		Flexor	3.0 (1.0–3.25)	1.0 (0.75–1.0)	1.0 (0–1.75)	0.010*	0.001*
MTS (R2-R1) (degree)	Elbow	Extensor	30.0 (10.0–45.0)	5.0 (0–37.50)	30.0 (0–68.75)	0.041*	0.094
		Flexor	30.0 (20.0–66.25)	15.0 (0–57.50)	40.0 (15.0–61.25)	0.034*	0.013*
	Wrist	Extensor	2.50 (0–20.0)	0 (0–3.75)	0 (0–10.0)	0.011*	0.050*
		Flexor	22.50 (10.0–33.75)	5.0 (0–20.0)	15.0 (7.50–25.0)	0.019*	0.264
	Finger	Extensor	0 (0–13.75)	0 (0–0)	0 (0–0)	0.109	0.061
		Flexor	20.0 (5.0–40.0)	5.0 (0–10.0)	10.0 (0–21.25)	0.034*	0.095

Values are presented as medians and interquartile ranges.

Abbreviations: *MAS* modified ashworth scale, *MTS* modified tardieu scale.

*P*^1^: statistical analysis with Wilcoxon signed ranked test, *P*^2^: statistical analysis with Friedman test.

*Significant at *p* < 0.05.

### Effect of BTxA injection on foot pressure analysis

**Figure 1:**
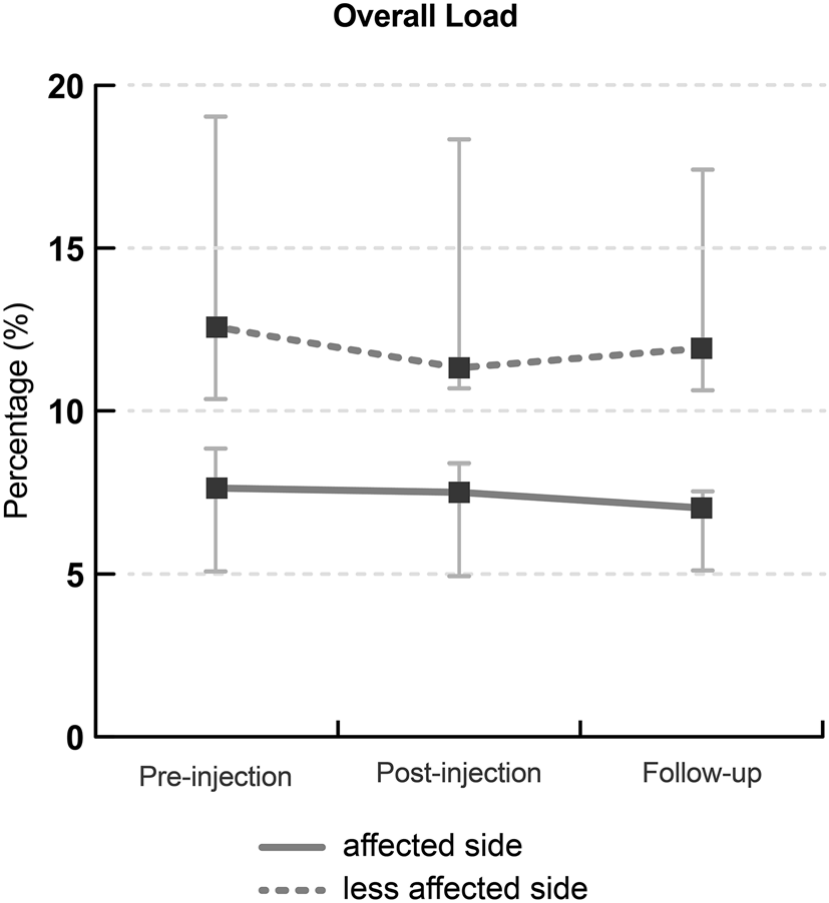
Percentage of overall load with foot pressure analysis at pre-, post- injection and follow-up evaluation separated into affected (solid line) and less affected (dashed line) sides. Values are median and inter-quarter ranges.

During walking, plantar pressure on the affected side after BTxA injection was significantly reduced in the post-intervention and follow-up evaluations (*p* = 0.020). This was reflected by the overall load, as presented in Fig. 1. Other spatiotemporal gait parameters were described in Table 3.

**Table 3:** Changes in foot pressure and postural balance analysis.

Parameters		Pre-injection	Post-injection (3 weeks later)	Follow up (3 months later)	*P* value	
					*P*^1^ (pre- post)	*P*^2^
Load	Overall load (%) (affected side)	7.64 (5.08–10.36)	7.51 (4.93–10.69)	7.03 (5.11–10.63)	0.363	0.020*
	Overall load (%) (less affected side)	12.57 (8.84–19.04)	11.32 (8.39–18.34)	11.92 (7.54–17.41)	0.638	0.204
	SI overall load	43.58 (21.77–62.87)	34.37 (20.19–59.42)	46.94 (19.77–59.42)	0.245	0.662
	SR overall load	0.64 (0.52–0.80)	0.71 (0.54–0.82)	0.62 (0.54–0.82)	0.177	0.662
Spatio-temporal parameters	Gait speed (m/s)	0.31 (0.14–0.51)	0.33 (0.13–0.51)	0.31 (0.13–0.49)	0.226	0.539
	Stride length (m)	0.51 (0.44–0.81)	0.56 (0.47–0.83)	0.55 (0.42–0.77)	0.169	0.211
	DLSD (s)	0.55 (0.45–1.98)	0.53 (0.45–1.72)	0.51 (0.45–1.65)	0.327	0.458
	SPD (s) (affected side)	1.03 (0.83–2.26)	0.97 (0.86–2.20)	0.97 (0.85–2.12)	0.807	0.869
	SPD (s) (less affected side)	1.34 (1.09–2.83)	1.33 (1.10–2.66)	1.42 (1.08–2.40)	0.925	0.872
	SLSD (s) (affected side)	0.45 (0.36–0.50)	0.43 (0.39–0.47)	0.44 (0.37–0.49)	0.245	0.775
	SLSD (s) (less affected side)	0.71 (0.55–0.92)	0.70 (0.65–0.86)	0.70 (0.57–0.82)	0.730	0.657
	SI SPD	18.73 (17.18–21.65)	19.46 (13.09–23.76)	18.56 (8.21–30.00)	0.331	0.835
	SR SPD	0.83 (0.80–0.84)	0.82 (0.79–0.88)	0.83 (0.74–0.92)	0.363	0.835
	SI SLSD	46.81 (35.31–68.46)	46.88 (33.51–77.94)	50.34 (31.72–63.01)	0.331	0.607
	SR SLSD	0.62 (0.49–0.70)	0.62 (0.44–0.71)	0.60 (0.52–0.73)	0.363	0.607

Values are presented as medians and interquartile ranges.

Abbreviations: *SI* symmetry index, *SR* symmetry ratio, *DLSD* double limb support duration, *SPD* stance phase duration, *SLSD* single limb support duration.

*P*^1^: statistical analysis with Wilcoxon signed ranked test, *P*^2^: statistical analysis with Friedman test.

*Significant at *p* < 0.05.

### Effect of BTxA injection on postural balance analysis

**Figure 2:**
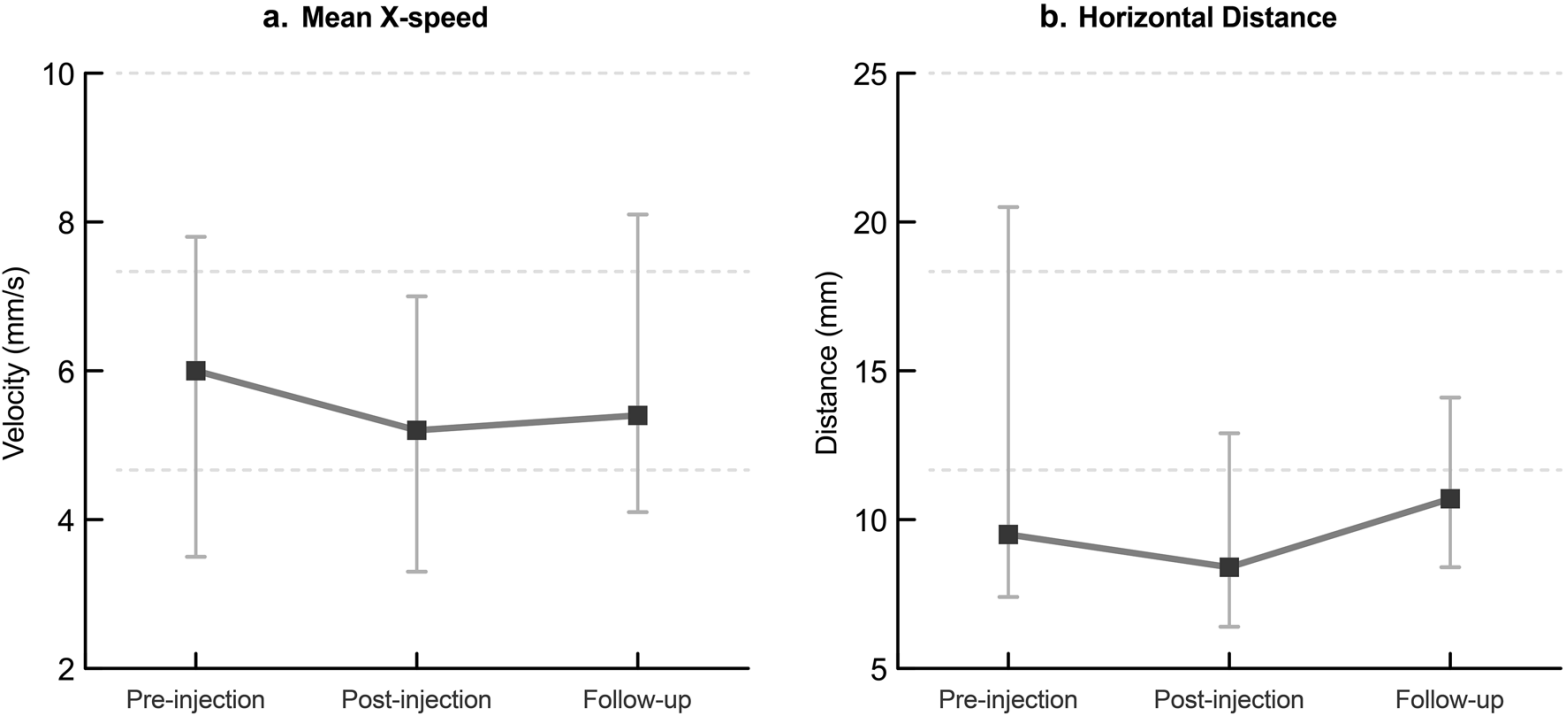
Postural balance analysis at pre-, post- injection and follow-up evaluation. (**a**) Velocity of mean X-speed; and (**b**) horizontal distance. Values are median and inter-quarter ranges.

Postural balance improved after BTxA injection in the hemiplegic spastic upper extremities. This was confirmed by the decreased balance parameters, including mean X-speed and horizontal distance in the eyes-opened state, as described in Fig. 2. Other balance parameters were listed in Table 4.

**Table 4:** Changes in postural balance analysis.

Parameters		Pre-injection	Post-injection (3 weeks later)	Follow up (3 months later)	*P* value	
					*P*^1^ (pre- post)	*P*^2^
Eye-opened	Mean X-speed (mm/s)	6.0 (3.5–7.8)	5.2 (3.3–7.0)	5.4 (4.1–8.1)	0.053*	0.223
	Mean Y-speed (mm/s)	8.4 (7.4–12.6)	8.4 (6.1–12.0)	9.6 (7.4–11.8)	0.118	0.135
	Mean VM (mm^2^/s)	15.2 (10.9–27.8)	13.6 (9.9–29.7)	17.4 (12.6–29.2)	0.173	0.319
	H distance (mm)	9.5 (7.4–20.5)	8.4 (6.4–12.9)	10.7 (8.4–14.1)	0.047*	0.296
Eye-closed	Mean X-speed (mm/s)	8.2 (5.1–11.2)	9.6 (5.4–11.0)	8.8 (6.9–12.3)	0.513	0.926
	Mean Y-speed (mm/s)	14.7 (10.9–23.6)	12.8 (10.4–20.0)	16.2 (10.7–22.3)	0.733	0.689
	Mean VM (mm^2^/s)	28.9 (19.9–56.9)	31.2 (21.1–44.5)	40.4 (28.3–67.2)	0.955	0.199
	H distance (mm)	10.3 (9.3–14.1)	11.9 (9.1–15.3)	12.4 (11.0–18.2)	0.910	0.204
Dynamic	Time (s)	98.7 (75.4–152.6)	81.9 (72.1–149.8)	88.6 (68.4–141.5)	0.248	0.301
	Total distance (mm)	5590.3 (4245.4–6613.0)	4846.0 (4125.8–5860.5)	4693.4 (4412.1–8571.1)	0.477	0.301
	ML distance (mm)	4209.5 (3549.5–5596.3)	3994.3 (2905.5–7640.1)	3186.9 (3089.0–8152.1)	0.722	0.741

Values are presented as medians and interquartile ranges.

Abbreviations: *VM* velocity moment,; *H* horizontal, *ML* mediolateral.

*P*^1^: statistical analysis with Wilcoxon signed ranked test, *P*^2^: statistical analysis with Friedman test.

*Significant at *p* < 0.05.

### Correlation between improvements in upperextremity spasticity and gait function

There was a positive correlation between the improvement in MAS score of the elbow flexor and increased stance phase duration (SPD) (s) on the affected side (r = 0.563, *p* = 0.020). There was also a positive correlation between the change in MTS (R2-R1) of the wrist extensor and decreased double limb support duration (DLSD) (s) (r = 0.562, *p* = 0.036). Furthermore, there was a strong positive correlation between the change in MTS (R1) of the finger extensor and the increased relative speed (1/s) (r = 0.753, *p* = 0.019), stride length (m) (r = 0.752, *p* = 0.020), and relative stride length (r = 0.770, *p* = 0.015).

### Correlation between improvements in upper-extremity spasticity and postural balance control

In the static study with eyes-closed state, the improvement in spasticity of the finger flexor was related to a change in the mean X-speed and mean Y-speed. There was statistically significant positive correlation between the improvement in the MAS score of the finger flexor and decreased mean X-speed (r = 0.603, *p* = 0.029). Additionally, there was a positive correlation between the change in MTS (R1) of the finger flexors and decreased mean Y-speed (r = 0.609, *p* = 0.027).

**Figure 3:**
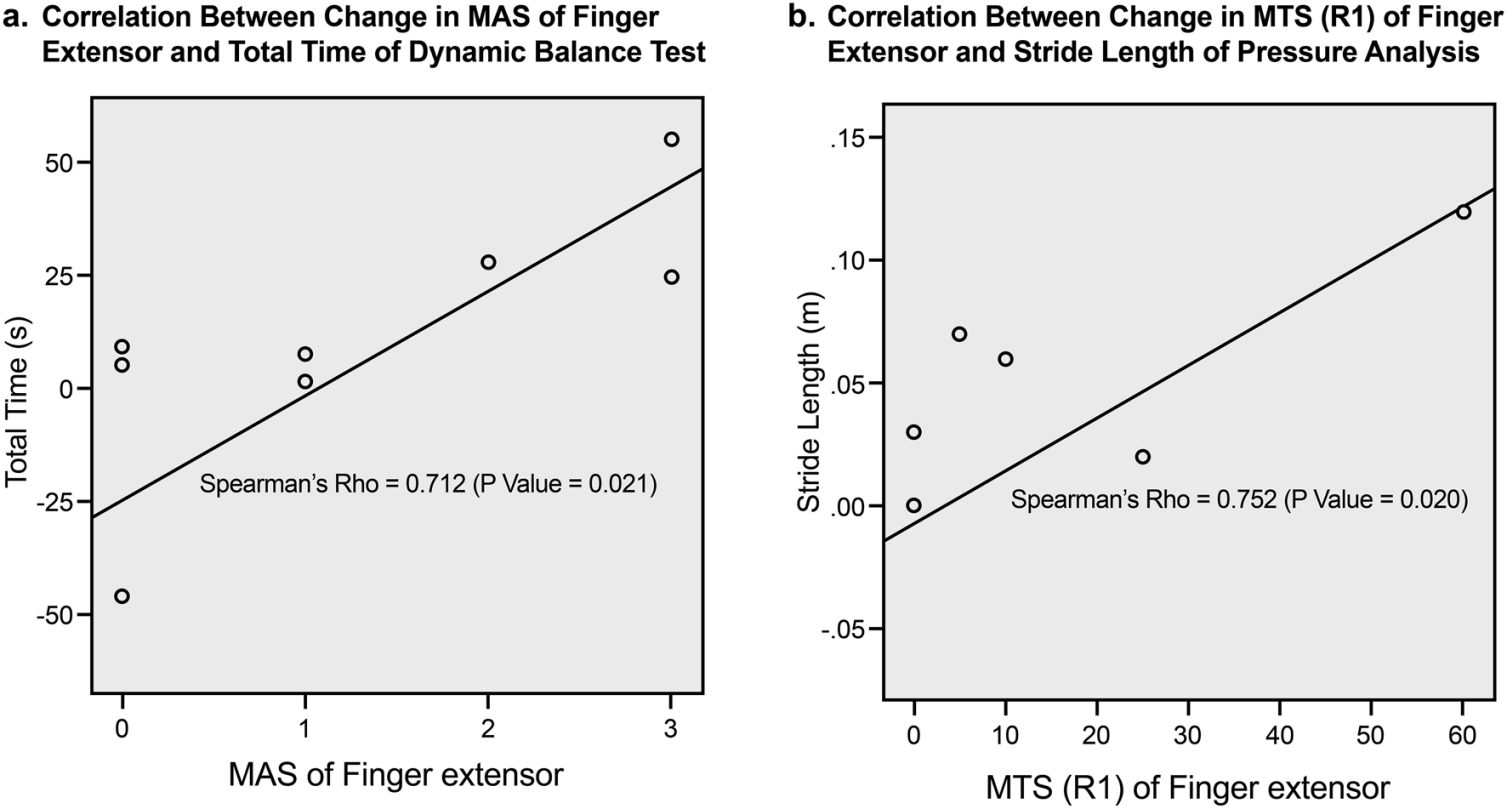
Scatter plots. (**a**) Example of correlation between improvement (pre- and post-injection) in upper extremity spasticity and postural balance parameter and (**b**) example of correlation between improvement (pre- and post-injection) in upper extremity spasticity and gait parameter. Values are mean and standard error of the mean.

In the dynamic study, the improvement in spasticity of the wrist and finger extensors had an impact on reducing the performing time, total distance, and horizontal distance taken to complete the task. There was a strong positive correlation between the improvement in MAS score of the wrist extensor and reduced performing time (r = 0.749, *p* = 0.008). Moreover, there was a significantly strong association between the improvement in MAS score of the finger extensor and reduced performing time (r = 0.712, *p* = 0.021) and horizontal distance (r = 0.712, *p* = 0.021). In addition, there was a strong positive correlation between the change in MTS (R1) of the finger extensor and the decreased total distance (r = 0.736, *p* = 0.038). Examples of scatter plot with strong correlations were presented in Fig. 3.

## Discussion

Upper-extremity spasticity can be an obstacle during standing and walking because it aggravates hemiplegic posture and may interfere with stable balance and safe gait. Thus, changes in the mechanical properties of muscle tissue components and spasticity in the upper extremities can lead to impaired balance and gait characteristics. We determined that BTx injection in the hemiplegic spastic upper-extremity has a positive effect on postural balance and gait function in stroke patients. Compared to the recent studies, our study quantitatively proved this hypothesis using plantar pressure and postural balance analysis. We also attempted to specify and present a possible explanation for the relationship between improvement in spasticity of the upper extremities and balance control with gait ability. Finally, as upper-extremity spasticity decreased, postural balance and gait function improved.

Along with spatiotemporal parameters, we considered the symmetry index and symmetry ratio^35^. We found a numerical improvement in these symmetric parameters; however, we failed to determine any statistical significance. Further large-scale studies are needed to diversify the severity of spasticity to determine a clear link between these two different results. Otherwise, the mean X-speed and horizontal distance in the eyes-opened test were significantly decreased at the post-intervention and follow-up evaluations compared to the baseline. In addition, descriptive finding showed that these two parameters were higher during follow-up evaluation than post-injection. The effect of BTx injection usually lasts three months, approximately^36^. As time passes, the efficacy of BTx on upper-extremity spasticity, which may affect trunk control, will decrease. Due to the drug mechanism of BTx, the value of the balance parameters can be deteriorated in follow-up evaluation.

We used mean X-speed, mean Y-speed, horizontal, vertical and total distance for postural balance analysis. Decreased speed and shortened distance meant that motion swing control was improved.

The MAS and MTS are evaluating tool for spasticity in the resting state, there has a limitation on assessing changes during performance. Efforts have been made to evaluate these aspects in a previous study; in particular, there was an attempt to analyze the angle of the elbow flexors during gait^23^. Even though we had a limitation on evaluating changes in spasticity with movement, we tried to resolve this problem by dynamic functional evaluation through balance and gait assessment.

Improvement in postural balance after BTxA injection was clearly presented on mediolateral distance, As a result, upper extremity BTx treatment might be helpful if there is a problem with horizontal direction control when performing balance and gait evaluation in patients with upper-extremity spasticity.A few studies have already investigated this subject and have focused on upper trunk posture. Previously, Hefter et al. demonstrated that injections of BTx into the affected arm of hemiplegic patients improved abnormal lateral trunk flexion. This shift of the center of mass of the upper body toward the midline improves various gait parameters, including faster gait speed, reduced pre-swing duration of both legs, and increased step length of the non-affected leg^37^.

In our study, the improvement in postural balance was significantly correlated with finger flexor spasticity. Post-stroke patients have their own spasticity on various location of upper limb. Participants who enrolled in this study had upper-extremity spasticity on wrist and finger dominantly. Spasticity on wrist and finger showed more improvement after BTx injection compared to those of shoulder and elbow. Because of this finding, we considered the correlation between postural balance and finger flexor spasticity was clarified.

Our study could not confirm the spatio-temporal gait parameters during post-injection, changes in upper-extremity spasticity were significantly correlated with spatiotemporal parameters such as DLSD, relative speed, stride length, relative stride length change, and SPD change on the affected side between pre- and post-injection. This might be due to small study population, which might have influenced the differences between the two types of analyses.

A recent systemic review suggested that instrumental and laboratory measures of gait improved after BTx injections in different muscle groups of the upper and lower extremities^24^. Gait changes were presented using various methods, including spatiotemporal, kinematics, kinetics, and electromyography. In particular, our study selected not only spatiotemporal parameters but also plantar pressure loading for gait measurements and additionally calculated gait symmetry. Furthermore, we considered a balance component which had significantly contributed to the overall walking function by further analysis of postural balance and confirmed the correlation between improvement in these parameters and improvement in upper-extremity spasticity.

This study has several limitations. First, an insole-type pressure analysis system has inherent limitations in measuring spatial parameters such as step length. Therefore, studies using other methods to examine the spatial parameters should be conducted. Second, there may be confounding factors such as age, sex, location of the stroke lesion, and functional aspects related to gait speed. Further research to investigate these confounding factors should be conducted to clarify the metrics of post-stroke gait. Third, the sample size was small. We expected that there would be a significant improvement in spatiotemporal parameters; however, the sample size was insufficient to determine the clear difference in the three points of evaluation. This finding should be supplemented by further studies.

## Conclusions

Our study suggested that improvement of spasticity on hemiplegic upper extremity has great impact on improvement of gait and balance function in stroke patients. We quantitatively evaluated each parameters and revealed the clear correlation between them.

## Data Availability

The datasets generated during and/or analyzed during the current study are not publicly available due to including patient's personal and sensitive information, but are available from the corresponding author on reasonable request.
